# Analyzing the Biochemistry of Saliva: Flow, Total Protein, Amylase Enzymatic Activity, and Their Interconnections

**DOI:** 10.3390/ijms26031164

**Published:** 2025-01-29

**Authors:** Jose A. Parraca, Alejandro Rubio-Zarapuz, José Francisco Tornero-Aguilera, Vicente Javier Clemente-Suárez, Pablo Tomas-Carus, Ana Rodrigues Costa

**Affiliations:** 1 Departamento de Desporto e Saúde, Escola de Saúde e Desenvolvimento Humano, Universidade de Évora, 7004-516 Évora, Portugal; ptc@uevora.pt; 2 Comprehensive Health Research Centre (CHRC), University of Évora, 7004-516 Évora, Portugal; 3 Faculty of Sports Sciences, Universidad Europea de Madrid, Tajo Street, 28670 Madrid, Spain; josefrancisco.tornero@universidadeuropea.es (J.F.T.-A.); vicentejavier.clemente@universidadeuropea.es (V.J.C.-S.); 4 Grupo de Investigación en Cultura, Educación y Sociedad, Universidad de la Costa, Barranquilla 080002, Colombia; 5 Departamento de Ciências Médicas e da Saúde, Escola de Saúde e Desenvolvimento Humano, Universidade de Évora, 7004-516 Évora, Portugal; acrc@uevora.pt; 6 Center for Sci-Tech Research in Earth System and Energy (CREATE) Universidade de Évora, 7004-516 Évora, Portugal

**Keywords:** saliva biochemistry, amylase activity, protein concentration, physical activity, non-invasive biomarker

## Abstract

This study examines the biochemical profile of saliva, focusing on flow rate, total protein concentration, and the enzymatic activities of amylase and catalase. The study aims to explore the correlations between these parameters and their response to physiological stress induced by physical activity, providing insights into saliva’s diagnostic potential. Thirty-one participants were recruited, and saliva samples were collected before and after a structured physical activity session. Biochemical parameters were analyzed using established protocols to assess changes induced by exercise. Significant positive correlations were observed between protein concentration and amylase activity, particularly in post-exercise conditions. No significant correlations were found between the salivary flow and enzyme activities. Catalase activity displayed a weaker association with protein levels. These findings suggest that saliva can be a non-invasive biomarker for systemic health and stress responses. The study highlights the diagnostic utility of saliva and underscores the need for further investigations in younger and healthier populations to broaden the applicability of these results.

## 1. Introduction

Saliva plays a critical role in both oral and systemic health, acting as a fundamental component in maintaining oral hygiene, facilitating digestion, and serving as a diagnostic tool for various diseases. It is composed primarily of water, but it also contains important organic and inorganic molecules that contribute to its protective functions, including buffering acids, remineralizing tooth enamel, and providing an antimicrobial action [1]. The production of saliva is influenced by daily rhythms, with peak secretion around meal times and minimal flow during sleep, highlighting its dynamic nature in response to physiological needs. Additionally, saliva’s diagnostic potential is underscored by its ability to reflect serum biomarkers, making it a valuable non-invasive medium for monitoring health conditions ranging from cardiovascular diseases to endocrine functions and infectious diseases [2]. Its ease of collection and the correlation of many salivary parameters with serum levels offer a promising avenue for the non-invasive diagnosis and monitoring of systemic health [3].

The relationship between salivary biochemical parameters, such as enzyme activities and protein concentration, and systemic health is critical for developing non-invasive diagnostic methods that integrate oral and general health monitoring [4]. Among these components, mucins, immunoglobulins, cystatins, defensins, histatins, lactoferrin, agglutinin, lysozyme, and lactoperoxidase play pivotal roles in oral health by facilitating processes such as digestion and protection against caries and by combating oral infections [5]. Each salivary gland—parotid, submandibular, and sublingual—contributes uniquely to the saliva composition, producing a mixture of serous and mucinous secretions essential for maintaining oral tissues and initiating the digestion process. Furthermore, saliva’s diagnostic potential extends beyond oral health, offering insights into systemic diseases through the transfer of biomolecules from the blood to the saliva via ultrafiltration and diffusion processes [6]. This capability positions saliva as a non-invasive, accessible diagnostic tool for monitoring various health conditions, including cardiovascular diseases, endocrine functions, and infectious diseases, thereby underscoring the significance of understanding saliva’s biochemistry and its relationship with physical health [7].

The intricate relationship between various components within saliva, including enzymes like amylase and catalase, reflects the body’s physiological and pathological states, influencing physical fitness and systemic health [8]. The activities of these enzymes not only play a critical role in the oral cavity’s defence mechanisms, but also serve as potential indicators of broader health issues [9]. For instance, variations in amylase activity have been linked to stress responses, whereas alterations in catalase activity could indicate oxidative stress levels, providing insights into the body’s antioxidative capacity [10]. The necessity for a holistic approach to health monitoring is increasingly recognized, with studies showing that salivary biomarkers can reflect both physiological and pathological states, enabling early diagnosis and targeted interventions [3,4]. These findings underscore the potential of saliva analysis as a valuable tool for comprehensive health assessments and disease prevention. Given the foundational role of saliva in both oral and systemic health, as well as its diagnostic potential, this article proposes a focused study on the relationship between salivary biochemical parameters—specifically, salivary flow, protein levels, and the activities of enzymes such as amylase and catalase—and their collective impact on physical health. By analyzing the interactions between salivary components and their associations with health outcomes, this study aims to establish saliva as a reliable, non-invasive diagnostic tool. This approach seeks to simplify health assessment methods, leveraging saliva’s accessibility for early detection, prevention, and management of various health conditions.

## 2. Results

**Figure 1:**
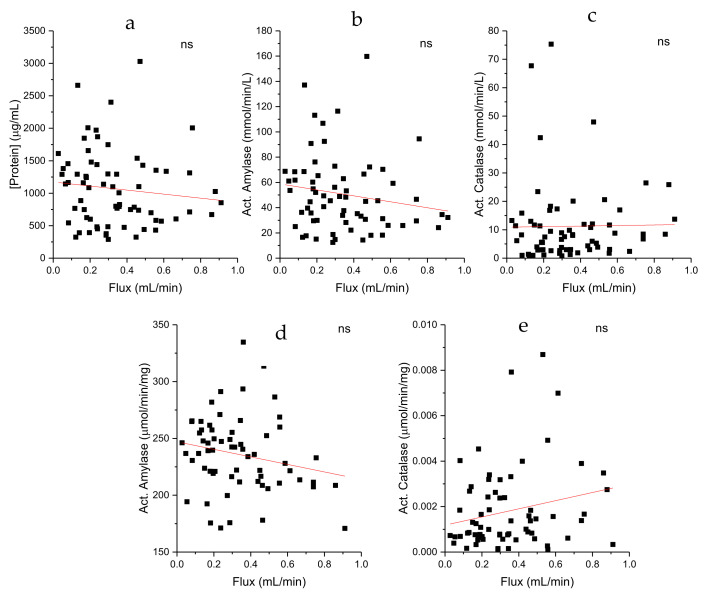
Relation among salivary flux, protein concentration, and enzymatic activities of amylase and catalase, expressed in mmol/min/L of saliva (upper panel). In the lower panel, specific enzymatic activities are represented (µmol/min/mg protein). ns means that the slope is not significantly different from 0. (**a**): flux and protein; (**b**): flux and act. amylase (mmol/min/L); (**c**): flux and act. catalase (mmol/min/L); (**d**): flux and act. amylase (µmol/min/mg protein); (**e**): flux and act. catalase (µmol/min/mg protein).

Enzymatic activities of catalase and amylase are expressed in function of salivary volume (upper panel) or in function of the total salivary protein concentration (lower panel) (Figure 1).

The scatter plot shows individual data points representing different salivary fluxes and their corresponding concentrations of protein or enzymatic activity levels. The red line represents the trendline of the data which mathematically describes the relationship between the two variables.

The results show that no correlations were observed, revealing that the salivary flow does not influence protein concentration or amylase or catalase activities. However, a tendency of the flux to influence negatively protein concentration, as well as amylase activity, can be observed, and, inversely, a tendency to positively influence catalase activity.

**Figure 2:**
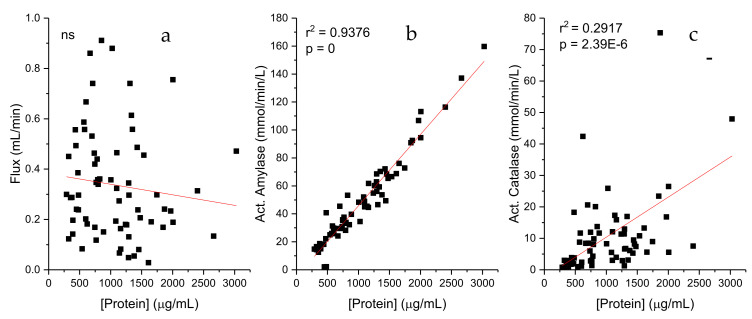
Correlation between protein concentration and salivary parameters: flux and enzymatic activities of amylase and catalase. The respective r^2^ and *p* values are shown. ns means that the slope is not significantly different from 0. (**a**): protein and flux; (**b**): protein and act. amylase (mmol/min/L); (**c**): protein and act. catalase (mmol/min/L).

Figure 2 illustrates the potential influence of total protein concentration (in µg/mL) on the enzymatic activities of amylase and catalase, both of which are expressed in mmol/L/min to ensure the independence of the variables. While no correlation was observed between the salivary flux and the protein concentration (as shown in Figure 1), positive correlations were identified for both enzymatic activities. For amylase activity, a strong linear relationship was observed (y = 0.0509x + 5.1176; R^2^ = 0.93764), indicating that amylase activity increases proportionally with higher protein concentrations. In contrast, the correlation for catalase activity was weaker (y = 0.01272x − 2.34173; R^2^ = 0.29169), suggesting a less consistent association. These results highlight the distinct behavior of the two enzymes in relation to protein concentration, with amylase showing a much stronger dependency. The R^2^ value suggests that approximately 93.76% of the variability in amylase activity can be explained by the protein concentration in this sample set.

**Figure 3:**
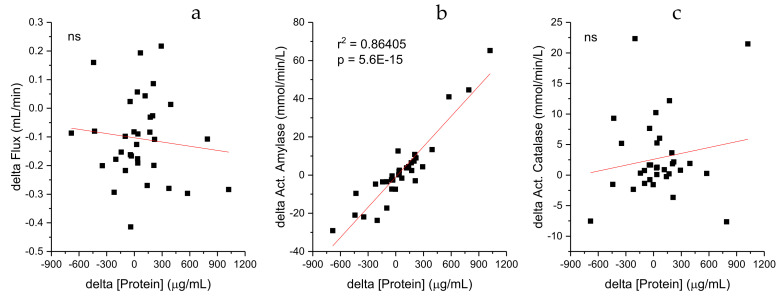
Correlation between the differences in the salivary parameters, post and pre physical activity (delta = post − pre). The respective r^2^ and *p* values are shown. ns means that the slope is not significantly different from 0. (**a**): delta protein and delta flux; (**b**): delta protein and delta act. amylase (mmol/min/L); (**c**): delta protein and delta act. catalase (mmol/min/L).

Exploring further the relation between salivary parameters and considering the positive correlations observed between protein concentrations and the amylase and catalase activities, Figure 3 seeks to correlate differences in parameters induced by the physical activity. The differences for individual parameters were calculated (delta = post − pre), and the scatter plot shows individual data points representing the delta for the concentrations of proteins and their corresponding differences in the other parameters (flux, amylase, and catalase activity). A positive correlation between protein concentration deltas and amylase activity deltas (act. amylase in mmol/L/min) was observed. The equation of the line (y = 0.0529x + 0.9363) and the coefficient of determination (R^2^ = 0.864) are displayed, indicating a strong linear relationship where the activity of amylase increases as the protein concentration increases. The R^2^ value suggests that approximately 86.4% of the variability in amylase activity changes are exercise-induced, a phenomenon which can be explained by the protein concentration changes being exercise-induced in this sample set. Although a positive correlation was observed between protein concentration and amylase activity (weak, but statistically significant), the differences in the variables induced by the physical activity were not correlated.

## 3. Discussion

Saliva plays an indispensable role in systemic health, acting as a diagnostic tool for a wide array of diseases [1]. In addition to the metabolites actively secreted into this fluid, contributing to its function in the digestive processes and in maintaining oral hygiene, its diagnostic potential is further underscored by the transfer of biomolecules from the blood to the saliva through ultrafiltration and diffusion processes [6], thereby reflecting the body’s physiological and pathological states and influencing physical fitness and systemic health [8]. Consequently, the aim of this study is to elucidate the intricate interconnections within salivary biochemistry, yielding profound insights into the biochemical dynamics of saliva.

Although there is a tendency for salivary samples with a lower flow to have a higher concentration of proteins, the salivary flow and total protein concentration in saliva are not correlated. This may be a consequence of the fact that the mechanisms that control the flow (greater or lesser amount of water in the saliva) and protein secretion (such as amylase, for example) are regulated by different pathways. Typically, the parasympathetic nervous system regulates the water volume in saliva and the sympathetic nervous system regulates secretion [11].

Amylase and catalase enzymatic activities are also not correlated with the salivary flow. However, the trends shown between the two enzymes are reversed. Amylase activity follows the same trend as that of protein concentration, while catalase follows the opposite trend. The explanation may lie in the fact that amylase is a protein actively secreted into the saliva in response to stimuli (namely adrenergic), constituting the predominant protein in saliva [12], while catalase is an intracellular enzyme which arises in saliva as a result of the passage of elements from the blood to the saliva, so a greater flow can facilitate this transference.

Enzymatic activities in saliva, such as those of catalase and peroxidase, play critical roles in maintaining oral health, as observed by Zahedani et al. 2017 [13]. Although catalase showed weaker correlations in this study, its activity underscores the complex biochemical dynamics of saliva and its potential implications for systemic health monitoring.

Regarding the relationships between the variables and the salivary protein concentration, a pronounced positive correlation between protein concentration and amylase activity in saliva samples under basal conditions was found, as evidenced by a high coefficient of correlation (R^2^ = 0.9376). This indicates that approximately 94% of the variability in amylase activity can be attributed to fluctuations in protein concentration. The equation of the trendline, y = 0.0509x + 51,176, quantifies this relationship, suggesting a consistent escalation in amylase activity with increasing protein concentrations. Subsequently, by examining the dynamic nature of salivary biochemistry in response to physical activity, it was demonstrated that exercise-induced changes in protein concentration and amylase activity are still strongly correlated. The trendline equation, y = 0.0529x + 0.9363, with an R^2^ value of 0.864 indicates a robust, yet slightly less pronounced, correlation compared to resting conditions. This variance intimates that physical activity modulates salivary biochemistry, potentially through mechanisms tied to the hydration status, blood flow, and hormonal fluctuations, thereby affecting protein concentration and enzyme activity [14]. The distinct correlation observed pre- and post-exercise elucidates the adaptability of salivary components to physiological alterations [15], shedding light on the body’s acute responses to stressors such as physical exertion. The biological implications of these discoveries are multifaceted. Amylase, a pivotal enzyme in starch breakdown [16], has been implicated in diverse physiological and stress-induced responses [17,18,19]. The observed correlation may reflect a broader systemic reaction, wherein protein levels in saliva—modulated by nutritional, metabolic, and stress-related factors—directly influence enzymatic activity [20]. This relationship holds the potential to serve as a biomarker for specific conditions or stress levels, offering a non-invasive portal into systemic health. The correlations identified between protein concentration and amylase activity in saliva suggest that these biomarkers might reflect not only systemic health, but also responses to various physiological stimuli, such as physical, emotional, or psychological stress. Amylase, being a stress-responsive enzyme [21], could potentially offer insights into sympathetic nervous system activity. Salivary amylase, as highlighted by Boehlke et al. 2015 [22], is a highly abundant enzyme with significant inter- and intra-individual variability. The strong correlation observed in this study between protein concentration and amylase activity underlines the enzyme’s potential as a marker for systemic and stress-related responses.

However, further research is needed to elucidate the underlying pathways linking physiological stressors and salivary biochemical markers, ensuring a more comprehensive understanding of these interactions.

Therefore, the robust correlations delineated in these findings underscore the potential of salivary analysis within clinical and research domains. By quantifying the relationship between protein concentration and amylase activity, this study augments the growing corpus of evidence advocating for the utilization of saliva as a diagnostic modality. The sensitivity of saliva to both resting and altered physiological states advocates for its utility in the non-invasive monitoring of health, stress responses, and, potentially, the efficacy of interventions aimed at ameliorating systemic health [23]. The strong correlation observed between salivary protein concentration and amylase activity in our study can be partly explained by the metabolic and physiological alterations associated with obesity. Obesity is known to activate the sympathetic nervous system and increase systemic inflammation, both phenomena of which are linked to elevated salivary amylase secretion as a stress-responsive enzyme [11,17]. Elevated salivary protein concentrations may further reflect systemic inflammation and oxidative stress often observed in individuals with obesity [8].

These findings reinforce the role of saliva as a sensitive medium for detecting systemic changes induced by obesity, particularly under physiological stress conditions such as physical activity. The dynamic response of amylase and protein concentration highlights their potential utility in assessing stress and metabolic status in obese populations.

These findings enhance the precision of current diagnostic methods by providing a broader understanding of an individual’s biochemical status, enabling early detection of conditions that affect salivary protein concentrations or enzymatic activities. Future research should include longitudinal studies to examine how these correlations evolve with chronic stress or disease progression and comparative studies to determine their applicability across diverse populations. Standardizing saliva collection and analysis methods, while addressing the specificity and sensitivity of these biomarkers, will be essential to fully realize the potential of salivary analysis in health monitoring. The strong correlations between protein concentration and amylase activity, both at rest and after physical activity, highlight saliva’s sensitivity to physiological changes. These findings support saliva’s potential for health monitoring and disease prevention, promoting its use in personalized medicine and clinical practice. As research advances, incorporating salivary biomarkers into diagnostics could revolutionize non-invasive health monitoring and provide innovative solutions for improving human health and well-being.

## 4. Materials and Methods

### 4.1. Participants

A total of 31 participants were enrolled in the study. The mean age and body mass index (BMI) of people were 51.3 (10.4) years and 31.5 (7.9) kg/m^2^, respectively. The convenience sample was recruited from the Lusitania family health unit in Évora (Portugal) and the University of Évora until April 2021. All procedures were conducted following the Helsinki Declaration (revised in Brazil, 2013) and approved by the University’s research ethics committee (GD/44902/2019).

Eligible individuals were females between the ages of 18 and 67 years who demonstrated the ability to walk independently without reliance on assistive devices (Table 1). However, participants were excluded if they were actively enrolled in other clinical studies, had previously undergone neuromodulation therapy, or had engaged in structured exercise programs within the six months preceding the study. Further exclusions applied to individuals who did not provide written informed consent, had undergone recent surgical procedures, or sustained musculoskeletal injuries within the past six months. Participants were also excluded if they presented with severe cardiovascular or respiratory conditions contraindicating exercise.

### 4.2. Training Procedure

Participants performed a strength training intervention for 1 h. Prior to the implementation of the strength training, participants completed a warmup consisting of the following exercises focused on motor control and activation of the abdominal section as a stabilizer:

- Bird dog: participants begin in a quadruped position on the floor, supporting their body on their palms and knees. From this starting position, they extend one arm forward and the opposite leg backward, maintaining balance and ensuring that the hips remain level and the abdominal region is engaged. The participants hold the extended position momentarily before returning to the starting position and alternating sides. The prescribed volume of work involves two sets of five repetitions per side, with 30 s of rest between sets, with an intensity equivalent to 50% of the repetition maximum (RM).
- Palof press: participants stand with the feet hip-width apart, facing perpendicular to an anchored elastic band held securely between their hands. The arms are extended in front of the chest with the shoulders flexed at a 90° angle. Maintaining a neutral spine, the participants perform a controlled squat while keeping the arms fully extended and the trunk aligned, resisting the rotational pull of the band. The exercise volume consists of two sets of five repetitions per side, with 30 s of rest between sets, performed at an intensity of 50% RM.

Once the warmup was completed, the participant started the strength training intervention composed of three free weight exercises (adapted push-ups, sit-to-stand, and glute bridge) and one banded exercise (standing one-hand row). Three sets of 10 repetitions for each exercise were performed with 1 min of rest between each set and exercise at an intensity of 70% RM, with each exercise being adjusted to reach this intensity for each participant [24].

- Adapted push-ups: depending on the participants’ capabilities, push-ups were performed as regular push-ups, with the participants only touching the floor with the palms of the hands and the toes, or as an adapted version, where the participants performed the exercise on their knees instead of their toes or against an elevated surface like a wall or a bench. Starting with the hands placed shoulder-width apart and the body forming a straight line from head to knees (or feet, if performed on a wall), the participants lower their chest toward the surface by bending the elbows, then push back to the starting position.
- Sit-to-stand: the participants begin seated on a chair or bench with the feet flat on the ground and hip-width apart. The participants rise to a standing position without using their hands, then return to the seated position. If necessary, weight was added to reach the required intensity
- Glute bridge: the participants lie on their back with knees flexed at a 90° angle with the feet flat on the ground and hip-width apart. Arms rest alongside the body. Then, by pressing through the heels, they lift their hips toward the ceiling, forming a straight line from shoulders to knees, then lower back down in a controlled manner. If necessary, weight was added to reach the required intensity.
- Standing one-hand row: a unilateral exercise. The participants stand with the feet hip-width apart, holding an elastic band securely in one hand, with the other end of the band anchored at a stable point at waist height. From this position, the participants pull the band toward the torso by bending the elbow, focusing on squeezing the shoulder blade toward the spine. The band is then returned to the starting position in a controlled manner. The resistance of the band was adapted to the fitness level of the participants to reach the required intensity.

The exercise protocol was designed specifically for this study, consisting of strength-based exercises adapted to the participants’ physical conditions. This protocol was adapted from previous studies involving fibromyalgia patients [11] but was tailored to the characteristics of the current cohort to ensure feasibility and safety.

### 4.3. Salivary Biomarkers

The salivary proteome presents unique opportunities for understanding systemic health, despite its inherent complexity and challenges in analysis, as noted by [25]. This study builds upon these perspectives by investigating specific biochemical components, such as protein concentration and enzymatic activities, to explore their diagnostic potential.

Unstimulated whole saliva was collected at rest and after exercise for each participant by direct draining into an ice-cold collection tube (pre-weighted) for 3 min. Subjects refrained from eating and drinking for at least 1 h before collection. After saliva collection, the tubes with the samples were weighted (for saliva flux evaluation, mL/min), centrifuged at 1.500× *g* for 10 min to remove food and cell debris, and the supernatants were stored at −20 °C until analysis. Prior to analysis, saliva samples were thawed on ice and centrifuged for 30 min at 4 °C and 13,000× *g* for the removal of mucinous material [26]. The supernatant total protein concentration was assayed using the Bradford method [27].

A dinitrosalicylic acid assay was used for measuring the starch-hydrolyzing activity of salivary α-amylase [28], minimized for 96-well microplates. The reaction mixture consisted of 1% starch solution and saliva sample (diluted to 10 µg protein/ml in 20 mM phosphate buffer (pH 7.0)). After incubation at 37 °C for 20 min, the reaction was halted by the addition of the DNS reagent. Samples were heated to 90 °C for 30 min. Further, sodium and potassium tartrate (40%) were added to the samples. Absorbance was measured at 530 nm. The absorbance values were then converted to glucose equivalents using a standard curve (3–18 mM). The results are expressed as µmol glucose/min/L saliva or as µmol glucose/min/mg salivary protein (specific activity).

Catalase activity was determined following the consumption of H_2_O_2_ at 240 nm [29], measuring absorbance every 30 s for 6 min. The reactional mixture consisted of salivary samples diluted in 10 mM potassium phosphate buffer (pH 7.0) and 0.2% H_2_O_2_. The enzyme activity is expressed in mmol of H_2_O_2_ consumed per minute per L of saliva or in µmol of H_2_O_2_/min/mg of salivary protein (specific activity).

For the purified catalase activity, glucose is not suitable as a standard in the DNS method, because salivary α-amylase primarily cleaves α-(1,4)-glycosidic bonds in starch or glycogen, producing maltose and oligosaccharides, not free glucose. However, in saliva, other enzymes such as maltase or isomaltase can break down maltose into glucose. Therefore, when measuring amylase activity in saliva, glucose can be used as a standard in the DNS method, as it reflects the total reducing sugars produced, including glucose formed indirectly through enzymatic action beyond amylase activity [30]. Other salivary peroxidases, such as myeloperoxidase (MPO), lactoperoxidase, and thyroperoxidase, can also contribute to the breakdown of hydrogen peroxide (H_2_O_2_) in saliva, complicating the specific measurement of catalase activity. These enzymes share the peroxidase activity, reducing H_2_O_2_ to water or oxygen and generating reactive oxygen species. In this study, we assume that the total peroxidase activity was measured. For future analyses, the selective inhibition of other peroxidases using KCN will be implemented to isolate catalase activity more accurately [31].

### 4.4. Statistical Analysis

All statistical analyses and graphics were conducted using Origin Pro 2017 SR2 (Origin Lab Corporation, Northampton, MA, USA). All statistical analyses were conducted using Origin Pro 2017 SR2 (Origin Lab Corporation, USA). Descriptive statistics were used to summarize the salivary parameters, including mean, standard deviation, and range. The relationships among salivary flow, total protein concentration, and enzymatic activities (amylase and catalase) were assessed using Pearson’s linear correlation analysis. The strength and direction of the correlations were quantified using Pearson’s correlation coefficients (r). To determine the statistical significance of the correlations, we tested whether the slopes of the regression lines were significantly different from zero using an analysis of variance (ANOVA). A significance level of *p* < 0.05 was applied to reject the null hypothesis. Additionally, R^2^ values (coefficients of determination) were reported to explain the proportion of variability in the dependent variable that can be attributed to the independent variable.

All statistical assumptions, including normality and homoscedasticity, were verified prior to performing the analyses. Scatter plots with trend lines and 95% confidence intervals were generated to visually represent the relationships and support the interpretation of the results.

## 5. Limitations and Future Perspectives

This study investigates the relationship between salivary biochemical parameters, such as protein concentration and enzymatic activities, in response to physical stress in a cohort of older and obese participants. While the findings provide valuable insights, the specificity of the sample limits the generalizability of the results. The lack of healthy or younger participants means that the applicability of these findings to broader populations remains uncertain.

Future studies should focus on validating these observations in diverse populations, including stratification by age, health status, and gender. Additionally, refining the methods for enzymatic activity analysis and exploring a wider range of salivary biomarkers will strengthen the diagnostic potential of saliva in systemic health monitoring.

## 6. Conclusions

This study demonstrates that salivary protein concentration strongly correlates with amylase activity, particularly in response to physical stress, suggesting that saliva can serve as a reliable, non-invasive biomarker of physiological and systemic health. While catalase activity showed weaker associations, it provides additional evidence of saliva’s complexity and diagnostic potential.

The results highlight saliva’s potential as a non-invasive tool for monitoring health and stress. This research lays the groundwork for its use in personalized medicine and preventive healthcare.

## Figures and Tables

**Table 1:** Characteristics of the participants (*n* = 31).

Participants (n)	31
Age (y)	52.6 ± 6.34
Weight (kg)	76.4 ± 13.3
Height (cm)	161 ± 5.78
BMI (kg/m^2^)	29.3 ± 4.38

Data are expressed as the mean ± SD (standard deviation) for quantitative variables. BMI: body mass index; n: number of participants; y: age; kg: kilogram; cm: centimeters; kg/m^2^: kilogram per square meter.

## Data Availability

All data is present on this article.
